# Transovarial transmission of DENV in *Aedes aegypti* in the Amazon basin: a local model of xenomonitoring

**DOI:** 10.1186/s13071-017-2194-5

**Published:** 2017-05-19

**Authors:** Cristiano Fernandes da Costa, Ricardo Augusto dos Passos, José Bento Pereira Lima, Rosemary Aparecida Roque, Vanderson de Souza Sampaio, Thais Bonifácio Campolina, Nágila Francinete Costa Secundino, Paulo Filemon Paolucci Pimenta

**Affiliations:** 1 Department of Environmental Surveillance, Health Surveillance Foundation of Amazonas State FVS-AM, Av. Torquato Tapajós, 6132, Colônia Santo Antônio, Zip 69.093-018 Manaus, Amazonas Brazil; 2 Universidade Nilton Lins, Programa de Pró Reitoria de Pesquisa e Pós-Graduação - UNICENTER. Laboratório de Entomologia Aplicada, Office 160, Av. Professor Nilton Lins 3259, Parque das Laranjeiras, Zip: 69 058-030 Manaus, Amazonas Brazil; 3 Laboratory of Physiology and Control of Arthropod Vectors - Oswaldo Cruz Institute – FIOCRUZ, Rio de Janeiro, Brazil; 4 Malaria and Dengue Laboratory, National Institute of Amazonian Research (INPA), Av. André Araújo, 2.936 Petrópolis, Manaus, Amazonas P.O. Box 2223, Zip 69080-971 Brazil; 5 René Rachou Research Centre, Oswaldo Cruz Foundation, Laboratory of Medical Entomology, Zip 30190-002 Belo Horizonte, Minas Gerais Brazil

**Keywords:** *Aedes aegypti*, Ovitraps, Transovarial transmission, Dengue, Xenosurveillance, Monitoring, Control

## Abstract

**Background:**

Transovarial transmission of dengue virus in *Aedes* spp. mosquitoes is considered an important mechanism for the maintenance of the virus in nature and may be implicated in the occurrence of outbreaks and epidemics of the disease. However, there are few studies involving transovarial transmission and viral vector monitoring as a surveillance tool and control strategy. The present study evaluated transovarial transmission of dengue virus in *Aedes aegypti* populations as a xenomonitoring strategy in municipalities of the Amazonas state.

**Results:**

*Aedes* sp. eggs (13.164) were collected, with 30% viability of third- and fourth-instar larvae. Transovarial transmission of DENV was detected in all municipalities. The transovarial infection rate (TOR) in the municipalities was 46% of the DENV positive samples. The minimum infection rate (MIR) was 17.7 in the state, varying from 11.4 to 24.1 per 1,000 larvae tested in the respective municipalities. Four DENV serotypes were identified, with DENV I and IV being present in all municipalities investigated. The number of reported dengue fever cases varied during this period.

**Conclusions:**

Our results suggest that transovarial transmission may be an important mechanism for the maintenance and spreading of the disease in Amazonas municipalities. Using qRT-PCR, it was possible to identify the four DENV serotypes in larval samples. The methodology used in the present study proved suitable as a DENV xenomonitoring model in immature mosquitoes, contributing to the development of systems for early detection of viral circulation and predictive models for the occurrence of outbreaks and epidemics of this disease.

**Trial registration:**

CAAE34025414200005015.

## Background

Dengue incidence has been high in recent years, and is now considered the most important arbovirosis in relation to public health, particularly in the tropical and subtropical regions of the planet, and is present in more than 140 countries [1, 2]. The most recent estimates indicate 390 million dengue infections per year of which 96 million require medical care or hospitalization, straining already precarious health systems [1]. *Aedes aegypti* is the main vector responsible for dengue in the Americas, and is found in over 70% of the municipalities in Brazil. *Aedes albopictus* is considered a secondary vector for the disease. Both are considered responsible for the transmission of chikungunya and Zika viruses as well [3–6].

Brazil is responsible for the highest number of dengue cases in the Americas [7]. Four DENV serotypes have been recorded in the country, with serotype frequency varying between regions [8]. The state of Rio de Janeiro was the first to register the simultaneous occurrence of three serotypes, DENV I, DENV II and DENV III [9]. However, in the past 20 years, the most significant event relating to the reintroduction and circulation of DENV serotypes in Brazil was the detection of DENV IV, in Manaus in 2008 [9, 10]. Since then, DENV IV was identified in Roraima in 2010, and São Paulo, Pernambuco, Bahia and Piauí in 2011 [10]. In 2011, the Amazonas state registered the largest dengue epidemic with the four viral serotypes circulating in the capital, Manaus, in which DENV IV was the most prevalent. However, there is no information concerning the serotypes circulating in the interior municipalities, due to the difficulty in transporting samples to the capital for viral isolation, which makes disease surveillance and control actions even more difficult.

Several authors have indicated that the identification of the circulating serotypes is fundamental for epidemiological studies and to identify factors associated with the progression of the disease as well as the clinical differences generated by the circulation of multiple serotypes [8, 11–15]. Three mechanisms of viral transmission in vectors have been described, the classical mechanism involving vector-host-vector, and sexual and transovarial transmission [16, 17]. Transovarial transmission, in which the females transmit the pathogens to their offspring, has been described for some arboviruses, including the dengue virus [18–20]. The role of transovarial transmission of dengue virus is still poorly understood and it is believed to be important for the maintenance of the virus in nature [21]. In 1992, Bosio and collaborators, in a laboratory study on vertical transmission of DENV in *Ae. albopictus* populations, showed that the populations tested were competent and were able to remain infected with the DEN-1 virus for several generations, allowing them to surpass the winter period and maintain the cycle during inter-epidemic periods [16]. Maintenance of the dengue virus in nature is known to be associated with the existence of an interaction virus-vector, that is, the susceptibility to infection and the capacity to transmit the pathogen, which can be detected in immature stages using molecular diagnosis techniques, thus allowing the knowledge and early surveillance of the risk of transmission of the disease [12, 17, 22].

Samples of both larvae and adult mosquitoes have shown great potential with respect to monitoring and identification of circulating dengue virus serotypes, which can enable viral monitoring in areas of difficult access once it is a methodology for viral monitoring in vectors [23]. The development of methods such as reverse transcription followed by real-time polymerase chain reaction (RT-PCR) has provided powerful tools for virus vector surveillance and epidemic risk assessment [24, 25]. Recent studies have shown that transovarial infection is not a rare occurrence in nature, and elevated rates of natural DENV infection have been observed, which may contribute to a better understanding of the role and mechanism of transovarial transmission in the spreading of epidemics, in addition to the early detection of DENV in vectors [12, 22, 26, 27].

In this context, the use of ovitraps to monitor vector spatial and temporal distribution has constituted an excellent tool, not only due to the low cost and ease of use, but mainly for their sensitivity and specificity in the collection of *Aedes* spp. eggs. Additionally, ovitraps are used to direct vector surveillance and control actions, in which they can be used for virus surveillance, particularly to monitor transovarial transmission and vectorial competence.

Continuous monitoring of the *Ae. aegypti* population, using tools capable of generating indicators related to the density of infestation and associated with the early detection of virus circulation may contribute to the development of epidemics prediction models for dengue [12, 24, 26, 28].

The present study aimed to identify the circulating DENV serotypes in larvae originating from eggs collected in ovitraps, in four municipalities of the Amazonas state as a proposition for the use of this tool as a strategy for xenomonitoring.

## Methods

### Study region

**Fig. 1:**
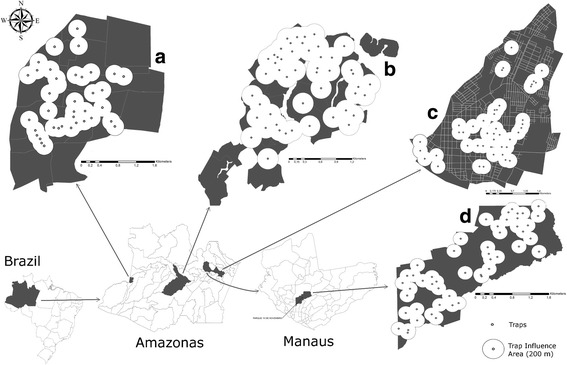
Map showing the geographical localization of the sites where egg collection was performed in municipalities of the Amazonas State: 3,538,387 inhabitants, emphasizing the grids selected to place the ovitraps. **a** Tabatinga (52,272 inhabitants). **b** Coari 75,965 (inhabitants). **c** Itacoatiara (86,839 inhabitants). **d** Manaus - AM (Parque 10 de Novembro neighborhood; 41,256 inhabitants)

Four priority municipalities of the Amazonas state were selected according to the Guidelines from the National Program for Dengue Control (PNCD); these were distributed over different regions: Coari situated in the Rio Negro and Rio Solimões region (40°5′6″S, 63°8′30″W), with a population of 90,605; Itacoatiara located in the Middle Amazonas (30°8′11″S, 58°26′33″W), with a population of 83,146; Tabatinga situated in the Alto Rio Solimões region (40°15′12″S, 69°56′19″W), with a population of 46,186; and Manaus situated in the Lower Rio Negro region, (30°6′26″S, 60°1′34″W), with the largest population at 1,731,993 (Fig. 1).

Urban, adjacent, and predominantly residential, non-verticalized areas were selected, in which autochthonous dengue cases were recorded in the previous 3 years and during the present study. Sample collection was performed between December 2013 and May 2014. Ovitraps were placed in previously selected neighborhoods that showed elevated rates of infestation, according to the rapid survey of *Aedes aegypti* infestation index (LIRAa) [28].

A total of 50 ovitraps were placed in each municipality, in inhabited residences, where they remained for three consecutive weeks with the paddles being replaced weekly, in which the eggs were collected, with the exception of the Manaus municipality where the ovitraps were set for only 1 week [29]. All the residents were instructed with regard to the study and signed the Free and Informed Consent Form (FICF) as approved by the Ethics Committee (CAAE N. 34025414200005015).

The traps were installed in peridomiciles, in shaded locations, protected from the sun and rain, at a maximum height of 1.5 m from the ground [12, 30]. The traps were monitored weekly by Endemics Control Agents from the municipalities that participated in the study, both in setting the traps, as well as collecting and forwarding the samples. The paddles were sent to the Entomology sub-management - SGENTO-FVS/AM in Manaus, where the eggs were counted and hatch took place. After eggs hatch, larvae were fed with fish foods until the L3/L4 stages and identified using a dichotomous key, according to Consoli and Lourenço-de-Oliveira [31]. *Aedes aegypti* larvae were separated into pools and used in the present study. Following that process, the larvae were cooled down to −20 ° C, washed 3 times in a saline buffered solution, placed in Eppendorf tubes, and transferred to a −70 °C freezer.

### Molecular analysis

RNA extraction was performed on pools of up to 30 *Ae. aegypti* larvae, including replicates for each sample, for the detection and amplification of DENV. RNA extraction was performed as described in [32]. Following centrifugation, 140 μl of supernatant was processed for extraction of viral RNA using the QIAamp® Viral RNA mini Kit (31 Qiagen, Hilden, Germany) according to the manufacturer’s instructions [33]. The samples were stored at −70 °C immediately after the extraction. qRT-PCR was performed using the Power SYBR® Green RNA-to-C_T_^TM^ 1-Step (Applied Biosystems, Foster City, USA) kit.

The primers used to amplify all the DENV were: forward: 5′-AGG AYA GAT GGT TAG AGG AGA-3′; reverse: 5′-CGY TCT GTG CCT GGA WTG AT-3′. These primers were designed based on the complete sequences of the four DENV serotypes, from partial capsid sequences and the 3′ noncoding region of DENV IV, isolated from different geographical regions in the world, according to Leparc-Goffart et al. [34].

qRT-PCR was performed in Micro Amp®Fast Optical 96 plates - Well Reaction Plate with Barcode (0.1 ml) (Applied Biosystems), in a total volume of 15 μl per well. The standard, samples, and positive control (RNA extracted from a mosquito infected with DENV II in the laboratory) were distributed in duplicate in the plate and the reaction blank (no template control, NTC), in quadruplicate. After adding all the components, the plate was sealed with Micro Amp® Optical Adhesive Film (Applied Biosystems). The reaction mix contained 7.5 μl of buffer, 0.3 μl of forward primer (10 pmol), 0.3 μl of reverse primer (10 pmol), 0.15 μl of polymerase enzyme, 3 μl of sample at 20%, and 3.75 μl of DEPC-treated water.

qRT-PCR reactions were processed in a 7500 Fast Real Time PCR Systems (Applied Biosystems) thermal cycler as described in [32]. The plasmid DNA was then digested with Faun DIN Restriction Enzyme (Life Technologies, Carlsbad, USA) for 4 h. The GFX-Illustra® kit (GE Healthcare, Little Chalfont, UK) was used to purify the digested DNA, according to the manufacturer’s protocol. The samples were quantified using the Nanodrop 2000 (Thermo Scientific, Waltham, USA). The curve built presented patterns (P) that varied between 300,000 and 3 DENV copies, whereby: P1 corresponded to 300,000 copies, P2 to 30,000, P3 to 3,000, P4 to 300, P5 to 30 and P6 to 3. The calculations were made according to the instructions provided in the Applied Biosystems manual [35].

### Construction of dengue virus serotypes curve

Serotyping assays for each serotype were performed under specific conditions, the PCR was performed using all four serotypes (DENV I, DENV II, DENV III and DENV IV) in cultures obtained from infected C6/36 *Ae. albopictus* cells and their specific primers. The following primers were obtained from the sequences of the serotypes DENV I, DENV II, DENV III and DENV IV: DENV I: 5′-ATA CCY CCA ACA GCA GGA ATT-3′ and 5′-AGC ATR AGG AGC ATG GTC AC- 3′; DENV II: 5′-GGA CCG ACA AAG ACA GAT CTT-3′ and 5′-CGY CCY TGC AGC ATT CCA A-3′; DENV II: 5′-AGA CGG GAA AAC CGT CTA TCA A-3′ and 5′-TTG AGA ATC TCT TCG CCA ACT G-3′; DENV IV: 5′-CCA TCC CAC CRA CAG CAG G-3′ and 5′-CAA GAT GTT CAG CAT GCG GC-3′ [34].

To determine the four different melting curves and to distinguish between all the viruses, the positive RNA used as standard was applied in a new assay in order to determine the serotype of the material collected in the field. Each sample was examined in triplicate using all the specific primers. The results obtained were analyzed according to the melting curve produced using the standard, based on the differences obtained for each serotype.

### Ovitrap indices

The eggs index obtained during the monitoring were calculated based on the ovitrap positivity index (OPI), which measures vectorial dispersal, the egg density index (EDI), which evaluates the mean number of eggs in positive traps, and the vectorial density index (VDI), which represents the mean number of eggs per trap set [12, 30].

### Transovarial transmission rate (TOT) and minimum infection rate (MIR)

The transovarial transmission rate was estimated taking into account the total number of positive pools *versus* the total number of pools analyzed and expressed as a percentage. The minimum infection rate in larvae was obtained from the total number of *Ae. aegypti* larvae divided by the total number of positive larvae pools × 1,000 [27, 36]. The results were compared to the data available in the literature and submitted to normality tests with a level of confidence of 95%.

### Dengue fever cases

The data regarding the dengue fever cases were obtained from the Information System for Notification of Disease (SINAN) for the period between 2011 and 2014, provided by the Foundation for Health Surveillance (FVS) of the Amazonas State Health Secretariat (SUSAM). The data were analyzed by epidemiological week, and the coefficient of incidence of reported cases (CI) was calculated for each municipality from the total number of reported cases divided by population and multiplied by 100,000 inhabitants. The classification of the coefficient of incidence of dengue was performed according to the criteria from the National Dengue Control Programme of the Brazilian Ministry of Health (PNCD/MS), whereby CI < 100 = low; 100 > CI < 300 = medium; and CI > 300 = high.

## Results

A total of 13,164 *Aedes* spp. eggs were collected during the course of this study; of these, 3,956 *Ae. aegypti* larvae were obtained, i.e. 30% of third- and fourth-stage larvae in relation to the number of eggs collected. Two hundred and forty-six *Ae. albopictus* larvae were collected, which were not included in this study. The egg indices varied between the municipalities. From the total number of traps set, 173 paddles contained eggs. Of these, 146 samples of *Ae. aegypti* larvae pools were obtained, and in 33 paddles containing eggs, no egg hatch or larval growth was observed. The lowest OPI was observed in Coari during the 12th epidemiological week at 20% trap positivity and the highest OPI was found in Tabatinga, during the 8th epidemiological week, with 58% positivity. The lowest EDI and VDI were also observed in Coari at EDI of 37.7 and VDI of 7.5, whereas the highest indices were observed in Tabatinga, with EDI of 131.5 and VDI of 58.0 obtained during the 10th epidemiological week. The number of larvae also varied between municipalities. A total of 1,223 larvae were obtained in Tabatinga, 1,120 in Itacoatiara, 871 in Coari, and 754 in Manaus (Table 1). The qRT-PCR was performed on 146 pools containing up to 30 individuals each. From these, 70 pools were found positive for DENV. Transovarial transmission of DENV was detected in all municipalities. Positivity of 46.3% was found, considering all samples evaluated. However, variation in the percentage of transovarial transmission was observed between the municipalities. Itacoatiara municipality showed the highest positivity percentage (57.8%), with the lowest positivity index being observed in Tabatinga (30.4%) (Table 2).

**Table 1:** Monitoring data and indices of eggs produced per ovitrap in Amazonas municipalities

	Epidemiological week	No. of installed palletes	No. of positive palettes	Total no. of eggs	OPI	EDI	VDI	Total no. of larvae
Amazonas		441	173	13,164	39	76.1	29.9	3,956
Manaus	51	50	20	1,812	40	90.6	36.2	742
Coari	10	46	15	1,146	33	76.4	24.9	242
	11	47	15	1,217	32	81.1	25.9	401
	12	50	10	377	20	37.7	7.5	228
	Total	143	40	2,740	28	68.5	19.2	871
Tabatinga	8	41	24	1,278	58.5	53.25	31.2	399
	9	43	21	1,944	48.8	92.6	45.2	490
	10	34	15	1,972	44.1	131.5	58.0	334
	Total	118	60	5,194	51	86.6	44.0	1,223
Itacoatiara	14	49	21	1,115	42.9	53.1	22.8	422
	16	39	20	1,196	51.3	59.8	30.7	343
	18	42	12	1,107	28.6	92.3	26.4	355
	Total	130	53	3,418	41	64.5	26.3	1,120

*Abbreviations*: *OPI* ovitrap positivity index, *EDI* egg density index, *VDI* vectorial density index

**Table 2:** qRT- PCR of *Aedes aegypti* larvae pools and DENV serotypes identified in Amazonas municipalities

	No. of analyzed larvae	No. of analyzed pools	No. of positive pools	% positive pools	MIR DENV	MIR serotypes	Serotypes DENV
Amazonas	3956	146	70	46.3	17.7	6.6	1, 2, 3, 4
Manaus	742	20	9	45	12.1	5.4	1, 3, 4
Coari	871	40	21	52.5	24.1	3.3	1, 3, 4
Tabatinga	1,223	41	14	30.4	11.4	4.1	1, 4
Itacoatiara	1,120	45	26	57.8	23.2	5.3	1, 2, 4

Regarding the minimum infection rate of the municipalities assessed, a MIR of 17.7/1,000 larvae was observed. When analyzing the data from the municipalities separately, it can be verified that the highest MIR was found in Coari at 24.1, and the lowest in Tabatinga, at 11.4. When only serotyped samples were included in the calculation, a MIR of 6.6 was obtained for all municipalities evaluated. The variation between the municipalities ranged from 5.4 in Manaus to 3.3 in Coari (Table 2). The presence of the four serotypes was detected in the serotyped samples, with DENV I and IV being found in all municipalities studied. Three serotypes were found in Manaus, Coari, and Itacoatiara, with DENV II only being detected in Itacoatiara (Table 2).

**Fig. 2:**
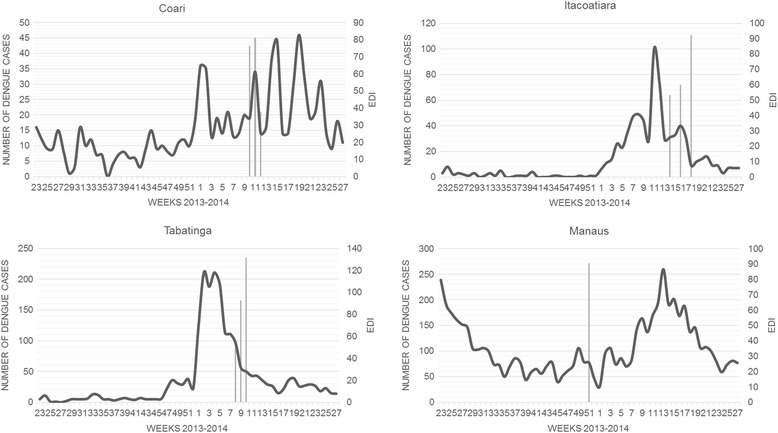
Reported dengue fever cases according to probable site of infection in the municipalities where the collection was performed, Amazonas (Brazil), between June 2013 and June 2014, by epidemiological week, and egg density index (EDI). *Legend*: Lines, number of dengue fever cases; Bars, egg density index (EDI)

Autochthonous dengue fever cases were recorded in the municipalities investigated, during the 3 years before the study, and in 2014, when samples were collected. Regarding the coefficients of incidence (CI) of reported cases, considerable variation was observed during the period evaluated (Table 3). According to the PNCD/MS classification, a dengue fever epidemic is registered in municipalities when CI is high and above 300/100,000 inhabitants. Using this criterion, dengue epidemics were registered in Coari (2011/2013), Itacoatiara (2011/2014), Tabatinga (2013/2014) and Manaus (2011/2013), demonstrating the intense DENV circulation in the municipalities evaluated (Table 3). During sample collection period (2013/2014), dengue fever cases were reported in all municipalities. The highest occurrence was registered in Tabatinga, with 202 cases of dengue fever between the 8th and 10th epidemiological weeks of 2014. Eighty and 65 cases were reported in Itacoatiara and Coari, respectively, during the collection weeks. Seventy-seven cases of dengue were registered in Manaus during the collection period, corresponding to the 51st epidemiological week of 2013 (Fig. 2). High egg density indices (EDI) were recorded for all municipalities during the sample collection weeks (Fig. 2).

**Table 3:** Historical series (2011–2014) of the coefficient of incidence (CI) rates of reported dengue fever cases per 100 thousand inhabitants in the selected municipalities

Municipalities	2011	2012	2013	2014
Coari	366.23	24.79	1,030.71	223.92
Itacoatiara	1,231.0	12.50	98.90	497.55
Tabatinga	250.58	224.83	653.88	1,724.68
Manaus	2,818.77	159.62	727.78	88.98

## Discussion

Dengue is endemic to the Amazonas state. According to Figueiredo et al. [29], the first disease cases were registered in 1998, associated with DENV I, and in 1999, DENV II was identified. DENV III was identified in 2002 and only in 2008 was DENV IV isolated from patient samples in Manaus, indicating the reintroduction of this strain in the country [10]. A study in which samples were collected from 432 patients of the Heitor Vieira Dourado Tropical Medicine Foundation in Manaus, and were analyzed by RT-PCR, showed that 137 were positive, with simultaneous identification of the four serotypes. Of the 137 samples, serotypes DENV II, DENV IV, DENV III, and DENV I were identified in 51, 31, 22, and 17 samples, respectively, which contributed to the authors suggestion that this disease is endemic to the capital [31].

The detection of transovarial transmission in vectors indicates that it is possible to identify dengue virus in *Ae. aegypti* mosquitoes during epidemic and inter-epidemic periods and that this transmission mechanism may be an important factor for the maintenance of DENV in nature [19]. In the present study, the data regarding samples obtained from immature mosquitoes in Manaus show the presence of three serotypes simultaneously - DENV I, III and IV - and a transovarial transmission percentage for DENV infection in 45% of the samples analyzed. In laboratory studies on transovarial transmission, researchers have demonstrated the persistence of DENV up to seven generations originating from infected matrices [19].

Viral monitoring in mosquitoes has suggested that transovarial transmission rates (TOT) occur at low levels in nature [20, 32, 33, 37]. In 2008, in Roraima, Zeidler et al. [32] performed an RT-PCR evaluation of 44 *Ae. aegypti* larvae pools containing 1,172 larvae and were unable to detect the presence of DENV, even though the collection took place in areas with elevated transmission of the disease. In 1998, a TOT study by Chow et al. [38] failed to detect positive samples from 53 pools containing 10 larvae each. These studies suggest that TOT rates are low in nature and virus persistence may be reduced. However, failure in detecting TOT must be evaluated, as it may occur frequently [19, 33]. Several factors can contribute to failure in detecting TOT, including low sensitivity of the tests, inappropriate methodology, and mosquito sample maintenance issues, among others [39]. In addition, Zeidler et al. [32] suggested that the method of viral detection in *Ae. aegypti* larvae is not the most suitable to estimate the risk of epidemics and that adult insects should be collected instead, providing increased sensitivity. However, in 2012, Guedes et al. [40] claimed that viral monitoring in immature stages of *Ae. aegypti* can be used to identify the circulating serotypes in naturally infected adults. Our results point to positivity of 46.3% on average, varying from 30.4% to 57.8%, demonstrating that advances in the molecular methods applied in this work can influence both the sensitivity and the specificity of this type of monitoring.

In a prospective study on DENV transovarial transmission in the urban area of Bangkok, Thailand, the authors found a MIR that varied between 0 and 24.4, and suggested that the methods for collection of adult mosquitoes are laborious and that during periods of low infestation, it is more suitable to collect immature mosquitos [27]. The MIR observed in the four municipalities of the Amazonas state was 17.7 on average, with variation between 11.4 and 24.1. The highest infection rate observed by Thongrungkiat et al. [27] was obtained 4 months before the peak of dengue transmission in humans, demonstrating the importance of viral monitoring in immature mosquitos, with a TOT percentage of 42% of the DENV-positive samples very close to that identified in our study (46.3%). Of these, 47.9% of the samples were positive for DENV IV; 13.4% for DENV III; 5.04% for DENV I; and 3.4% contained DENV II. Of the serotypes identified in the present study, 47.4% were DENV I, 38% were DENV IV, 9.5% were DENV II, and 5.1% of the samples were DENV III, a result that is in accordance with the data previously quoted [27].

Conversely, in a laboratory study performed on mosquitoes, in Florida, an infection percentage of 11% was observed in *Ae. albopictu*s and of 8% in *Ae. aegypti,* using qRT-PCR [41]. Data obtained from a study on vertical transmission, in Belo Horizonte-MG, Brazil, showed that 37.4% of the tested samples were positive for DENV. Among the *Ae. aegypti* larvae tested individually, DENV II was present in 21.4% of the samples. DENV II and I co-infection was identified in 6.7% of the samples. Co-circulation of DENV II and III was also observed in 6.1% of the samples evaluated [32]. Viral co-infection of *Ae. aegypti* larvae by more than one serotype was demonstrated for the first time in Brazil by these authors. The MIR observed by those authors was 13.8, revealing an elevated infection rate in the vectors as identified in the present study.

Variation between the municipalities was observed when analyzing the coefficient of incidence, CI, of dengue cases. Elevated CIs were observed in Manaus in 2011 and Tabatinga in 2014, characteristic of dengue epidemics; however, the circulating serotypes were not identified from the fever cases. A study performed in the city of Cuiabá, Mato Grosso state-Brazil, found a MIR of 10.5 obtained from *Ae. aegypti* larvae, where DENV IV was identified. Following genetic sequencing analysis of DENV, the authors showed similarity to the DENV IV genotype II strain from Manaus [42]. This is an important result because it demonstrates the origin and geographical distribution of the strains that circulate in the country. Although the values of positivity and minimum infection rate found are considered high, future studies should consider performing viral culture in order to demonstrate its viability.

The data obtained in the present study were similar to DENV vertical transmission studies performed in different countries and in Brazil, as previously mentioned. Our results suggest that transovarial transmission may be elevated in areas where the disease is transmitted, which may contribute not only for the maintenance of DENV in nature, but also for the amplification of the cases, since the adult insects are born naturally infected. qRT-PCR was shown to be an important tool for the diagnosis and identification of DENV serotypes present in immature mosquitoes. Several protocols have been produced, which facilitated the use of this technique, reducing eventual errors that may occur in this type of analysis. A novel fact provided by our data is that for the first time, it was possible to evaluate the serotypes circulating in municipalities of the interior of the state of Amazonas, without the need for new technology and without involving high costs for sample transportation. The detection of more than one circulating serotype in immature mosquito populations suggests the imminent risk of occurrence of more serious disease cases. As such, our work reveals an opportunity to gain a greater understanding of the role of vertical transmission of DENV in vectors, which can be considered a key point for the early detection of the occurrence of dengue fever cases in a certain area.

## Conclusions

The results obtained were compatible with the literature data, demonstrating that the methodology used was suitable, and that it can be incorporated as a strategy for surveillance and viral monitoring in vectors. From viral monitoring in vectors, it is possible to generate control measures and amplify the monitoring system for the disease in risk areas. Egg sample collection using ovitraps confirms the potential of these traps as a monitoring tool for the vectorial and viral populations, with the possibility of early detection of the circulation of the virus before reaching epidemic proportions, which may contribute to more efficient and effective control measures. The results also show that it is possible to identify DENV viral serotypes using qRT-PCR in *Ae. aegypti* immature mosquitoes, which can also contribute to viral monitoring, especially in areas that are difficult to access or that do not possess the technology to store the samples for viral isolation. It is important to highlight the implications for future studies on vectorial competence and virus-vector interaction, as well as the mechanisms involved in the maintenance of dengue virus in nature. In this sense, we believe that the main contribution of this study resides in the development of a more suitable monitoring system for the early detection of viral circulation and the risk of epidemics and severe forms of the disease.

## Abbreviations

CI: Coefficient of incidence of reported cases
DENV: Dengue virus
EDI: Egg density index
FVS/AM: Fundação de Vigilância em Saúde do Amazonas
LIRAa: Rapid survey of *Aedes aegypti* infestation index
MIR: Minimum infection rate
NTC: No template control
OPI: Ovitrap positivity index
PNCD: Programa Nacional de Controle da Dengue
qRT-PCR: Real-time quantitative reverse transcription polymerase chain reaction
RT-PCR: Real time polymerase chain reaction
SGENTO: Department of Entomology
SINAN: Sistema de Informação de Agravos de Notificação
TOT: Transovarial transmission rate
VDI: Vectorial density index
